# Correlation between US-FNAC with BRAF V600E Mutation Analysis and Central Neck Lymph Node Metastasis in cN0 Papillary Thyroid Cancer

**DOI:** 10.1155/2021/9937742

**Published:** 2021-07-01

**Authors:** Ruoxuan Li, Jiping Hao, Zhao Zhu, Xudong Qiao, Ling Wang, Zubang Zhou

**Affiliations:** ^1^Department of Ultrasound Medicine, Ninth Hospital of Xi'an, Xi'an 710054, China; ^2^Department of Ultrasound Medicine, Gansu Provincial Hospital, Lanzhou 730000, China

## Abstract

The aim of this study was to explore the correlation between ultrasound-guided fine-needle aspiration cytology(US-FNAC) combined with BRAF V600E mutation analysis and central neck lymph node metastasis in cN0 papillary thyroid cancer, so as to provide reliable molecular evidence to use it for preoperative evaluation, operation procedure design, and postoperative follow-up planning in clinic. Specimens were obtained from 250 patients with cN0 thyroid cancer (TI-RADS≥4a, highly suspected of PTC by US-FNAC) after bilateral thyroidectomy and central neck lymph node dissection with accessible postoperative pathologic results of PTC and central neck lymph nodes and used for cytological diagnosis by H&E stain and BRAF V600E mutation detection. Single-factor analysis showed that differences between the central neck lymph node metastasis and nonmetastasis groups were statistically significant in gender, BRAF V600E mutation, and extracapsular extension. Logistic multivariate regression analysis showed significant differences in gender, BRAF V600E mutation, and extracapsular extension. Positive BRAF V600E mutations by US-FNAC, extracapsular extension, and male gender are risk factors of central neck lymph node metastasis in cN0 PTC metastatic PTC to central neck lymph node. Patients with those factors should undergo prophylactic central neck lymph node dissection.

## 1. Introduction

Thyroid cancer is one of the most common malignant tumors in the head and neck. According to Global Cancer Statistics 2018, thyroid cancer ranks in the ninth place in the incidence rate [1], 80%-90% [2] of which was attributable to papillary thyroid cancer (PTC), the most common subtype. Although ultrasonography is the preferred imaging examination for thyroid nodules, its sensitivity to examine central neck lymph node metastasis was low [3], while a closed correlation has been identified between metastasis to central neck lymph nodes and recurrence of cancer. Therefore, it is important to identify whether there is neck lymph nodes metastasis, especially for central neck lymph nodes before the surgery, which determines the prognosis of a patient as well as the method and range of the surgery. In this study, we enrolled 250 patients with cN0 PTC to receive ultrasound-guided fine-needle aspiration cytology (US-FNAC) combined with BRAF V600E mutation detection, so as to identify the potential application value of US-FNAC with BRAF V600E mutation detection in assessing central neck lymph node metastasis in cN0 PTC, in order to provide reliable molecular evidence to use it for preoperative evaluation, operation procedure design, and postoperative follow-up planning in the clinic.

## 2. Materials and Methods

### 2.1. Subject

We enrolled 250 patients with cN0 PTC who visited the ultrasound medicine department of Ninth Hospital of Xi'an and Gansu Provincial Hospital from Oct. 2017 to Feb. 2021, including 101 males and 149 females, aged 22-72 years (average age 44.8), and divided into the metastatic group (140 cases) and nonmetastatic group (110 cases) according to the postoperative pathologic results of central neck lymph nodes. Enrollment criteria are as follows: TI-RADS≥4a, highly suspected of PTC with US-FNAC, no central neck lymph node metastasis found by all preoperative examinations, PTC diagnosed by postoperative pathology, and accessible pathologic results of central neck lymph nodes. US-FNAC procedures of all subjects were satisfactory with successful cytologic examinations and BRAF V600E mutation detections. For all patients, thyroid nodes were found for the first time, with normal thyroid function, normal past conditions, and no history of radiation exposure. All participants signed the informed consents. Patients who met all the above criteria were enrolled in this study.

### 2.2. Instruments and Methods

#### 2.2.1. Instruments

Instruments are as follows: Color Doppler Ultrasound GE LOGIQ E9, linear array probe 9 L, 6 ~ 8 MHz, aspiration biopsy needle, CL type,23G × 50 mm. The BRAF gene mutation detection kit (PCR sequencing) (produced by Da An Gene Co., Ltd. of Sun Yat-sen University) was used.

**Figure 1:**
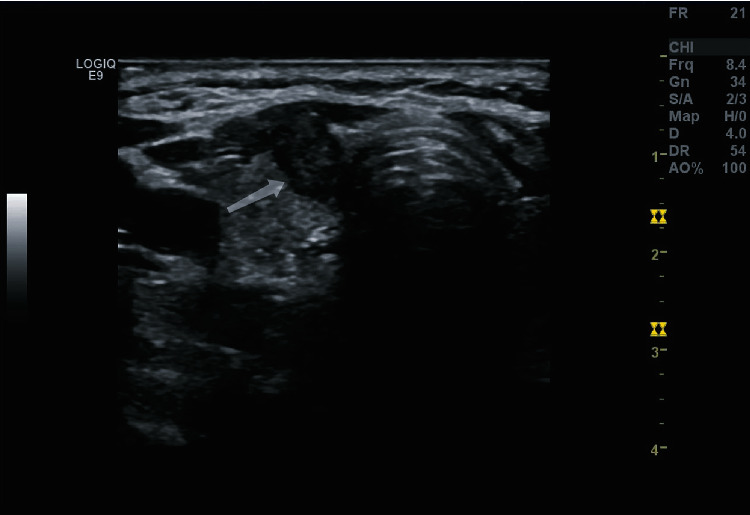
PTC ultrasonic image: the lesion manifests hypoecho, clear margin, inner microcalcification, a height-to-width ratio > 1, and extracapsular invasion.

This study assessed thyroid nodes according to TI-RADS in ATA guidelines, where the malignant signs include hypoechoic or extreme hypoechoic, unclear margin, a height-to-width ratio > 1, microcalcification (≤2 mm in diameter with punctate hyperechoic foci in this study), thyroid extracapsular invasion (an interrupted echo band of thyroid capsule caused by malignant nodule invasion, or an indistinct echo band of thyroid capsule without normal tissue between the malignant nodule and the capsule in ultrasound imaging), and neck lymph node metastasis, see Figure 1.

#### 2.2.2. US-FNAC and BRAF V600E Mutation Detection

**Figure 2:**
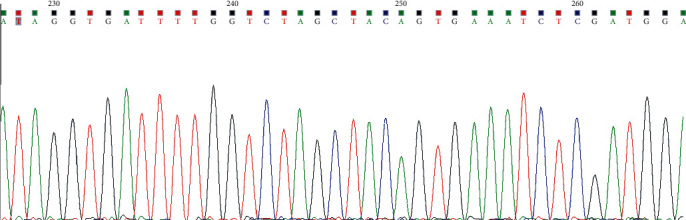
Negative results of US-FNAC combined with BRAF V600E mutation by PCR (no mutation).

**Figure 3:**
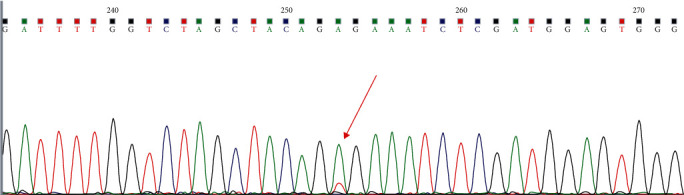
Positive results of US-FNAC combined with BRAF V600E mutation by PCR (see mutation site indicated by the arrow).

Biopsies were obtained from the node of TI-RADS≥4a; if multiple lesions, biopsies were obtained from the one most highly suspected of malignancy. US-FNAC was performed by skillful senior doctors. All participants underwent necessary examinations before puncture to make sure no contraindications for puncture and signed informed consents of invasive examinations. Patients were asked to lie on their back with neck hyperextension to sufficiently expose the puncture sites. After routine disinfection and laying towel, 5 ml lidocaine was used for local anesthesia. Under the guidance of ultrasound, the needle was inserted into the suspicious node repeatedly to acquire enough tissue, part of it was used for cytological diagnosis, and the rest stored in a pathological sample bottle at 2°C-8°C. After DNA extraction, BRAF V600E gene mutation was detected by real-time fluorescent quantitative polymerase chain reaction (PCR) (see Figures 2 and 3). After the postoperative pathologic results obtained, the BRAF V600E gene mutation results in the metastasis group and the nonmetastasis group were observed, and differences and correlations in BRAF V600E mutation, clinical manifestations, and ultrasonic features in the two groups were analyzed.

### 2.3. Statistical Analysis

All statistical analyses were performed using SPSS 25.0 software. *χ*^2^ test was used for enumeration data and single-factor analysis. Multivariate logistic regression analysis was used for relative risk factors with a two-sided test. *P* values of less than 0.05 were regarded as statistically significant.

## 3. Results

### 3.1. Clinical Presentations and Ultrasound Image of Patients with cN0 PTC

In the 250 typical PTC patients, 40.4% males and 59.6% females, aged 22-72 with 115 cases < 45 years old and 135 cases ≥ 45 years old where there were 112 cases with multiple lesions and 138 cases with single lesion and 114 cases with lesions ≥ 10 mm in diameter and 136 cases less than 10 mm. In addition, there were 129 cases with positive results of US-FNAC combined with BRAF V600E mutation detection while 121 cases negative, 140 cases with central neck lymph node metastasis while 110 cases without, 99 cases where ultrasound images showed clear margins while 151 cases with indistinct margins; 146 cases with a height-to-width ratio > 1 and 104 cases with a height-to-width ratio < 1; 101 cases with microcalcification while 149 cases without, and 105 cases with malignant extracapsular invasion while 145 cases without.

### 3.2. Correlation between Neck Lymph Node Metastasis and Clinical Presentations and Ultrasonic Features in cN0 PTC Patients

The single-factor analysis showed statistically significant differences in gender (*χ*^2^ = 18.489, *P* ≤ 0.001), BRAF V600E mutation (*χ*^2^ = 38.193, *P* ≤ 0.001), and extracapsular invasion (*χ*^2^ = 40.987, *P* ≤ 0.001) between the central neck lymph node metastasis group and the no central neck lymph node metastasis group (see Table 1). No significant differences were found in the other variants, so gender, BRAF V600E mutation, and extracapsular invasion were enrolled into the subsequent multivariate logistic regression analysis.

According to the results of multivariate logistic regression analysis, central neck lymph node metastasis was significantly influenced by gender (*P* = 0.002), BRAF V600E mutation (*P* ≤ 0.001), and extracapsular invasion (*P* = 0.001) (see Tables 2 and 3).

## 4. Discussion

Preoperative determination of neck lymph node metastasis in PTC patients is closely related to the patient condition assessment and prognosis. There have been a large body of research on the diagnostic efficiency of ultrasound in neck lymph node metastasis where it is reported that the sensitivity of two-dimensional ultrasound in the diagnosis of central neck lymph node metastasis is only 12.1% [4], and there were about 60% of PTC patients whose neck lymph node metastasis had not been found before the operation [5]. However, views on surgical methods remain divided, i.e., whether prophylactic neck lymph node dissection is needed. In the 2015 American Thyroid Association Management Guidelines for Adult Patients with Thyroid Nodules and Differentiated Thyroid Cancer, prophylactic neck lymph node dissection is not recommended for patients with T1, T2, and cN0 stages [6], while it is still controversial whether the operation scheme is applicable to all patients. For example, Chinese scholars like Liu et al. [7] believed that there was still a lack of evidence to routinely perform such operation for all patients. However, Hwang and Orlof [8] believed that routine central neck lymph node dissection could block the metastasis of thyroid cancer to the lateral neck lymph nodes, so as to improve the prognosis. Nevertheless, prophylactic neck lymph node dissection may lead to multiple complications, such as recurrent laryngeal nerve injury and temporary or permanent hypoparathyroidism. Therefore, it is of great significance to identify neck lymph node metastasis in PTC patients before operation, especially the central neck lymph nodes, so as to reduce surgery-caused trauma, develop a scientific follow-up system, and improve patients' quality of life.

In recent years, there have been many studies on biomarkers of PTC, particularly in BRAF gene [9]. However, it has always been controversial whether there is a correlation between BRAF V600E mutation and neck lymph node metastasis. For example, Cohen et al. [10] found that BRAF V600E mutation is closely related to neck lymph node metastasis in PTC patients, while Hong et al. [11] believed that BRAF V600E gene mutation detection results of FNAC-obtained thyroid node samples could not effectively predict central neck lymph node metastasis and thyroid extracapsular invasion in PTC patients. In this study, we have done a single-factor analysis of central neck lymph node metastasis, clinical manifestations, and ultrasonic features in PTC patients of the cN0 stage, which showed that central neck lymph node metastasis is related to gender, BRAF V600E mutation, and extracapsular invasion. Further, multivariate logistic regression analysis of all above risk factors showed that central neck lymph node metastasis was significantly influenced by gender, BRAF V600E mutation, and extracapsular invasion in ultrasonic images. Among them, analysis in BRAF V600E mutation showed that the *β* value of 0.941 indicated that BRAF V600E gene mutation had a positive effect on neck lymph node metastasis, and OR of 3.836 indicates that with positive BRAF V600E gene mutations, the risk of central neck lymph node metastasis was 3.836 times higher than negative mutations with other independent factors unchanged. Similarly, we found that the risk of central neck lymph node metastasis in male patients was 2.564 times higher than that in female, and the risk in patients with thyroid extracapsular invasion was 2.956 times higher than that in patients with intact capsule, suggesting that BRAF V600E mutation was not the only cause of central neck lymph node metastasis, so that it cannot be used independently to predict the risk of central neck lymph node metastasis but can just be used as a reference indicator of neck lymph node metastasis. Therefore, other independent indicators to predict the risk of central neck lymph node metastasis in patients cN0 stage PTC should be found to improve the assessment of metastasis risks, which is consistent with the findings of Hong et al. [11] and Zhang et al. [12]. In our previous study [13], we found that female gender, BRAF V600E gene mutation, and extracapsular invasion were risk factors for central neck lymph node metastasis in PTC patients, and logistic regression analysis showed that central neck lymph node metastasis was caused by BRAF V600E gene mutation and extracapsular invasion in combination, which is slightly different from the results of this study, due to the expanded sample size of the latter. Meanwhile, the PTC patients enrolled in this study were not limited to those with classic PTC pathological types and were all in the cN0 stage, while in the previous study, the sample size was smaller, PTC pathology enrolled were all classic types, and patients were those with no central neck lymph node metastasis only found by preoperative ultrasound examination.

## 5. Conclusion

Comprehensive and systemic evaluations are needed by taking US-FNAC combined with BRAF V600E mutation detection, extracapsular invasion in ultrasonic images, and gender into consideration for patients with thyroid nodes of TI-RADS≥4a, highly suspected of malignancy, and without neck lymph node metastasis by all preoperative examinations to assess the risk of central neck lymph node metastasis so as to avoid adverse effects caused by excessive surgery. Male patients with BRAF V600E mutation and extracapsular invasion have a higher risk of central neck lymph node metastasis, so prophylactic central neck lymph node dissection should be performed at the same time of lesion resection.

## 6. Limitations

The study still has limitations that patients enrolled were of a single race (all Chinese Han nationality), while a previous report [14] has found that BRAF V600E mutation may be related to race in PTC patients. Therefore, subsequent studies should enroll patients of multiple nationalities to further explore the correlation between US-FNAC combined with BRAF V600E mutation detection and central neck lymph node metastasis in patients with cN0 PTC.

## Figures and Tables

**Table 1:** Correlation between central neck lymph node metastasis and clinical presentations and ultrasonic features.

	Central neck lymph node metastasis	No central neck lymph node metastasis	*χ* ^2^	*P*
Gender				
Female	100	49	18.489	≤0.001
Male	40	61		
Age				
<45	58	57	2.677	0.102
≥45	82	53		
Tumor diameter				
<10 mm	76	60	0.002	0.967
≥10 mm	64	50		
Tumor number				
Single	82	56	1.462	0.227
Multiple	58	54		
BRAF V600E mutations				
Negative	92	29	38.193	≤0.001
Positive	48	81		
Extracapsular invasion				
No	106	39	40.987	≤0.001
Yes	34	71		
Height-to-width ratio				
<1	54	50	1.201	0.273
>1	86	60		
Microcalcification				
No	87	62	0.854	0.355
Yes	53	48		
Margin				
Unclear	85	66	0.013	0.909
Clear	55	44		

**Table 2:** Assignment description.

Factor	Variable	Assignment description
Central neck lymph node metastasis	*Y*	0: no central neck lymph node metastasis; 1: central neck lymph node metastasis
Gender	*X*_1_	0: female; 1: male
BRAF V600E mutation	*X*_2_	0: negative; 1: positive
Extracapsular invasion	*X*_3_	0: no; 1: yes

**Table 3:** Multivariate analysis of risk factors of central neck lymph node metastasis.

	*β*	Standard error	Wald	*P*	OR	95% CI
Gender	0.941	0.309	9.282	0.002	2.564	(1.399,4.698)
BRAF V600E mutation	1.345	0.313	18.422	≤0.001	3.836	(2.076,7.089)
Extracapsular invasion	1.084	0.315	11.867	0.001	2.956	(1.595,5.476)

## Data Availability

The data used to support the findings of this study are available from the corresponding author upon request.
